# Syngenetic rapid growth of ellipsoidal silica concretions with bitumen cores

**DOI:** 10.1038/s41598-021-83651-w

**Published:** 2021-02-19

**Authors:** Hidekazu Yoshida, Ryusei Kuma, Hitoshi Hasegawa, Nagayoshi Katsuta, Sin-iti Sirono, Masayo Minami, Shoji Nishimoto, Natsuko Takagi, Seiji Kadowaki, Richard Metcalfe

**Affiliations:** 1 University Museum, Nagoya University, Chikusa, Nagoya, Japan; 2 Graduate School of Environmental Studies, Nagoya University, Chikusa, Nagoya, Japan; 3 Faculty of Science and Technology, Kochi University, Kochi, Japan; 4 Faculty of Education, Gifu University, Yanagido, Gifu, Japan; 5 Institute of Space-Earth Environmental Research, Nagoya University, Nagoya, Japan; 6 Nagoya City Science Museum, Nagoya, Japan; 7 Faculty of Science, Nagoya University, Nagoya, Japan; 8 Quintessa, Videcom House, Newtown Road, Henley-on-Thames, Oxfordshire, UK

**Keywords:** Planetary science, Geochemistry

## Abstract

Isolated silica concretions in calcareous sediments have unique shapes and distinct sharp boundaries and are considered to form by diagenesis of biogenic siliceous grains. However, the details and rates of syngenetic formation of these spherical concretions are still not fully clear. Here we present a model for concretion growth by diffusion, with chemical buffering involving decomposition of organic matter leading to a pH change in the pore-water and preservation of residual bitumen cores in the concretions. The model is compatible with some pervasive silica precipitation. Based on the observed elemental distributions, C, N, S, bulk carbon isotope and carbon preference index (CPI) measurements of the silica-enriched concretions, bitumen cores and surrounding calcareous rocks, the rate of diffusive concretion growth during early diagenesis is shown using a diffusion-growth diagram. This approach reveals that ellipsoidal SiO_2_ concretions with a diameter of a few cm formed rapidly and the precipitated silica preserved the bitumen cores. Our work provides a generalized chemical buffering model involving organic matter that can explain the rapid syngenetic growth of other types of silica accumulation in calcareous sediments.

## Introduction

Spherical or ellipsoidal, isolated silica concretions occur throughout the world in calcareous sedimentary rocks of widely varying geological ages. These concretions are harder than the surrounding rock due to being more highly enriched in SiO_2_ and typically have sharp boundaries^1,2^. Owing to their morphological and material characteristics, silica concretions have fascinated both geologists^3^ and non-scientists who have been motivated to consider how such concretions could have formed. Silica concretions also had a critical role in human history as they provided suitable raw material (commonly known as flint) for making stone tools, which was a fundamental industry in the lives of Paleolithic foragers, Mesolithic hunter-gatherers and Neolithic farmers^4,5^.

Previously, isotopic analyses have been used to understand the diagenetic processes that occurred during sediment burial and the formation of silica concretions^6,7^. Some of the concretions contain well-preserved fossils suggesting the possibility of involvement of biogenic processes during concretion formation^2,8–10^. Such silicification with preservation of delicate skeletal materials^11^ and silicified peats^12^ have also been reported and suggest that silicification occurred rapidly to preserve the detailed textures inside. Indeed, the occurrences of fossils in silica concretions have been widely known among archaeologists through their observations of prehistoric stone artifacts made of flint^13^. However, although many studies of silica concretions have been carried out over several decades, questions still remain regarding the syngenetic formation process and the rate of concretion growth^3,14^. In particular, why do many concretions have spherical shapes and sharp boundaries? and why does the localized enrichment of SiO_2_ eventually stop and the concretion stop growing? These questions are basically related to the mass transport processes and the concretion growth rates in the sedimentary matrix. However, this relationship has not been described precisely.

In a general sense, commonly observed enrichment of Si to form silica concretions has been explained by diffusion accompanied by silica super-saturation in the pore-water^3,15^. A related process is diffusion dominated, three dimensional migration of aqueous solutes when cementation conditions are isotropic as recognized also in many carbonate concretions^16–20^. Carbonate concretions that are morphologically similar to silica concretions have been found to contain organic material at their centres and micro-organisms, especially chemosynthetic anaerobes, have been shown to play an important role in concretion formation^21–25^. These processes give rise to a spherical or ellipsoidal morphology in relatively homogeneous sediments.

Carbonate concretions often exhibit analogous features to those observed in silica concretions: oval shapes, sharp boundaries, constant cement content and uniform stable isotope composition throughout the concretion body. Raiswell and Fisher^19^ suggested that such features are readily explained by a pervasive model of concretionary growth and this model is now widely accepted. However, they note that these features can also be explained by a diffusive growth model and that it can be difficult to distinguish the two models from observations made on concretions. Bojanowski and Clarkson^26^ proposed a diffusive model driven by chemical gradients to explain siderite concretions developed around central organic remains. Such concretions can be considered analogous to silica concretions developed around organic-rich cores. Based on these models for analogous carbonate concretions, both pervasive and diffusive growth models need to be considered as possible explanations for such silica concretions. In this paper we present detailed analyses of silica concretions from the Eocene Green River Formation in Utah, U.S.A. in order to shed light on their nature and rates of the processes involved in their formation.

## Geological setting and studied materials

Lower to middle Eocene lacustrine organic-rich carbonate and siliceous mudstone deposits of the Green River Formation are well-known oil source rocks^27–30^ and the process of bitumen formation in them due to burial diagenesis has been well described^31,32^. The organic-rich carbonaceous formation is widely distributed in the central United States, such as in the Greater Green River Basin in Wyoming, the Uinta Basin in northern Utah, and the Piceance Creek Basin in Colorado^33,34^. The thickness of the Green River Formation varies in each basin, but is about 900 m thick in the Indian Canyon section. Deposition of the lacustrine sediments occurred over a period of 9 Ma, between ca. 52 and ca. 43 Ma^34,35^. Based on the vitrinite reflectance of about 0.5–0.6 %Ro in the Indian Canyon section^29,30^, the maximum estimated burial depth of the formation is about 200–600 m. The maximum geothermal temperature is estimated at about 100 °C, which corresponds to the bitumen generation stage^36^.

**Figure 1:**
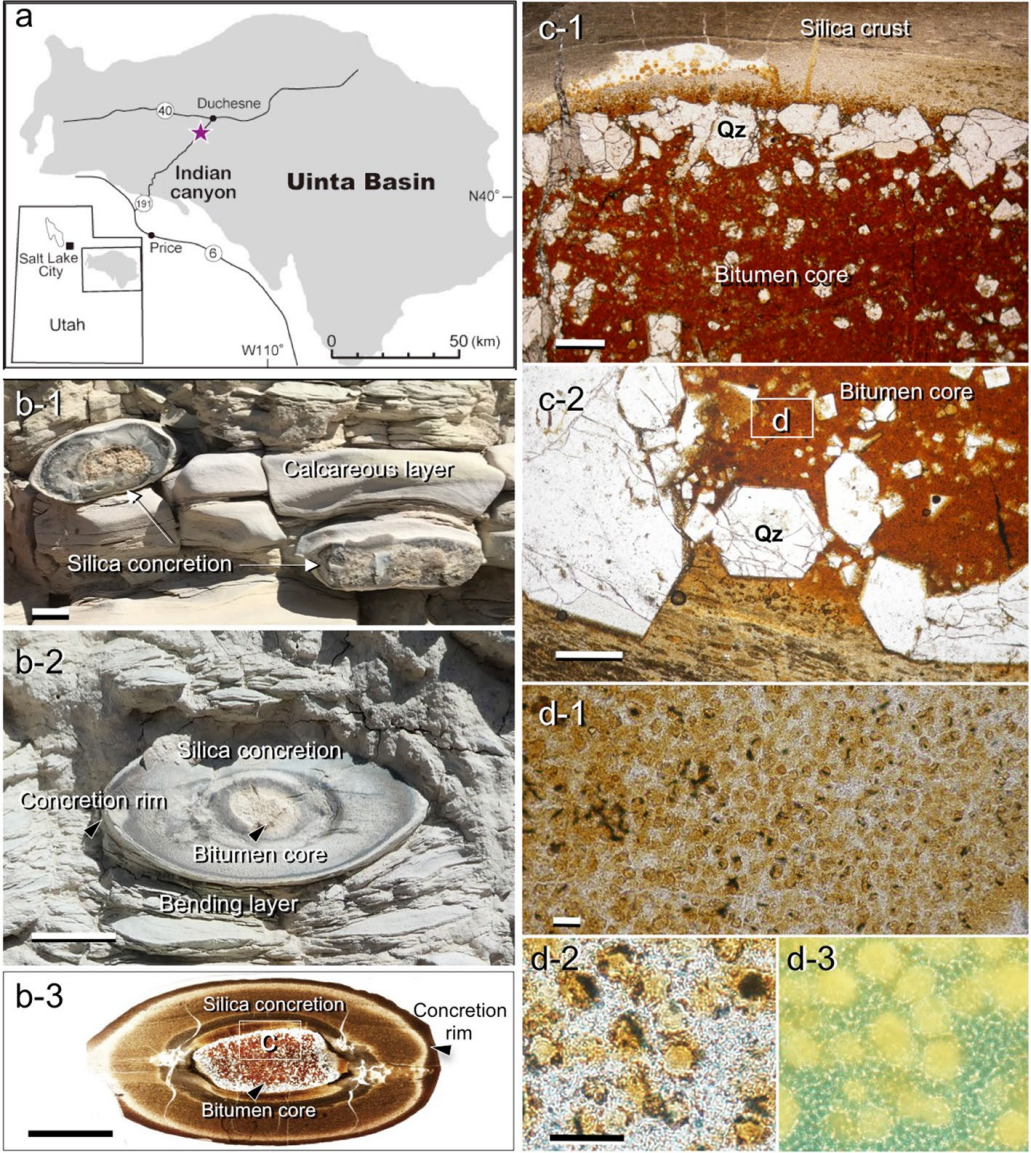
Occurrence of silica concretion with bitumen core. (**a**) Location map showing the sampling point in Green River Formation in the Indian Canyon section, western Unita Basin. (**b-1–2**) Occurrence of a silica concretions and the surrounding calcareous rock showing sedimentary layers bending around a concretion, but undeflected layers preserved inside a concretion (**b-3**). The terms used to describe parts of the concretions are also shown here (**b-2,3**). (**c-1,2**) Photomicrograph of a bitumen core and silica rich concretion containing euhedral quartz (Qz) crystals. (**d-1–3**) Dense accumulation of organic microspheres in the bitumen core. Well-preserved organic spheres observed under a microscope with plane polarised light (**d-1,2**) and by fluorescence microscopy (**d-3**). Scale bars in (**b**) is 1 cm, (**c**) is 0.5 mm and (**d**) is 0.1 mm. All photographs shown here were taken by H. Yoshida and R. Kuma.

Within the Green River Formation, there are facies dominated by calcareous sediments with intercalated bedded cherts. Silica concretions are mainly found in the upper part of the formation in the Indian Canyon section, western Uinta Basin (Fig. 1a). The Indian Canyon section is subdivided into six stages based on the lithology^37^; the fluvio-lacustrine, fluctuating deep-lake, stable lake, evaporation-dominant, fluctuating shallow-lake, and fluvio-lacustrine stages in ascending stratigraphic order. Previous studies revealed that silica-rich beds of the Green River Formation are characterized by the enrichment of Si, Mg, and Na^38,39^ and were deposited in a highly alkaline (pH > 9), saline-lake palaeo-environment^37^.

**Figure 2:**
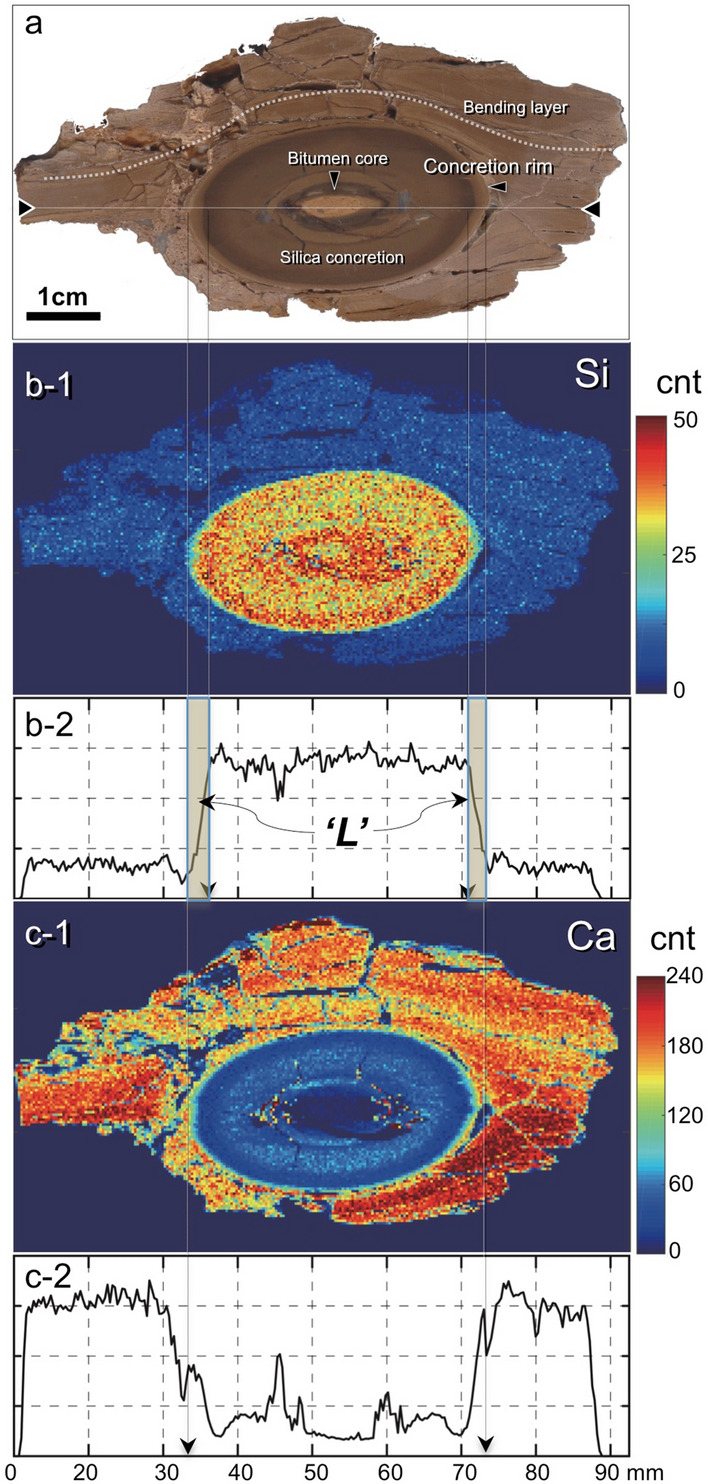
Si and Ca distributions in and around a silica concretion. (**a**) View of a plane cut through the centre of a silica concretion and the surrounding sedimentary rock. (**b,c**) Si (**b-1,2**) and Ca (**c-1,2**) distribution maps and concentration profiles (dotted line) across a silica concretion measured by SXAM (cnt: X-ray intensity; count per second). ‘L’ shown in the Si profile (**b-2**) is the width of the reaction front developed during concretion formation. Photograph (**a**) shown here is taken by H. Yoshida.

**Figure 3:**
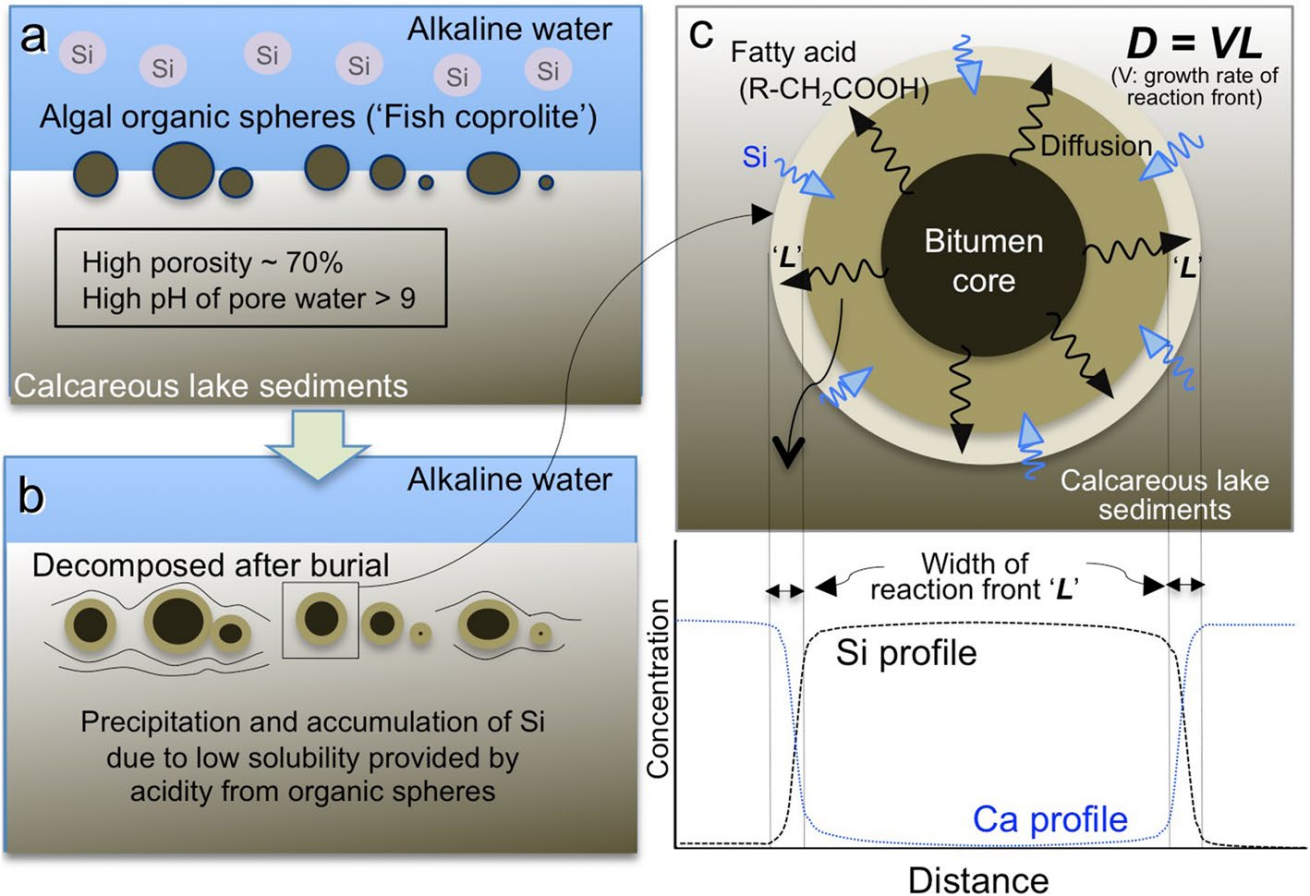
Formation process of silica concretion. Schematic illustration of the process of silica concretion formation after burial of algal organic spheres to form bitumen cores in calcareous lake sediments. (**a**) Concentric algal organic spheres initially grew in calcareous sediments in a shallow and highly alkaline lake rich in dissolved Si. (**b**) Subsequently, the algal organic spheres, probably ‘Fish coprolite’, decomposed and the pH of pore water in the sediment around the organic spheres decreased. The decreased pH of the pore waters then caused the abundant dissolved silica to precipitate around the decomposed organic spheres. (**c**) Conceptual view of the features of ellipsoidal silica concretions and relevant elemental (Si, Ca) profiles. The Si profile across the concretion formed in rather stable conditions under which solutes diffused continuously, driven by pH buffering and associated silica precipitation.

Ellipsoidal silica concretions with bitumen cores are observed in the carbonate-rich fine sedimentary rocks and occur mainly in the fluctuating shallow-lake stage. The axial dimensions of the concretions vary from 3 to 6 cm and all are readily distinguished from the surrounding calcareous layered rock by their sharp boundaries. The bitumen cores of all concretions are brownish to dark coloured and also ellipsoidal in shape with longer axial dimensions of 1 –2 cm in the centre (Figs. 1b-1–b-3, Supplementary Fig. S1).

## Results

All silica concretions are very hard and are laterally elongated parallel to the fine layers of the calcareous sedimentary rocks (Figs. 1b-1,b-2, 2a). The bitumen core of each silica concretion is surrounded by a dense silica enriched zone which is well preserved and readily identified in the surrounding layered sedimentary rocks (Figs. 1b-1,b-2). The terms for the parts of a concretion used in this text are also shown in Fig. 1 (b-2, b-3, Supplementary Fig. S1). Sedimentary laminae in the surrounding rocks are bent around the concretions (Fig. 1b-2, 2a), but horizontal sedimentary layers are identified within the concretions (Fig. 1b-3: showing sedimentary layers in a concretion). These features show that the concretions were probably hardened before compaction of the sediment was completed and therefore must have formed in an early stage of diagenesis. Within the concretions, chemically distinct parallel zones occur around the bitumen core (Fig. 2, Supplementary, Fig. S4) and suggest the concretion grew outward from the core.

SEM examination of the concretion between the bitumen core and the concretion rim, are composed of fine (c. 10 µm) silica crystals which are equidimensional and show no significant variation in size and shape across the concretion (Supplementary Figs. S2a–d). A very small number of very fine rhombohedral dolomite crystals probably pre-date the concretion^40^ and became incorporated in the silica that formed the concretion, with no dissolution occurring (Supplementary Fig. S2).

A combination of results from XRD and XRF analyses can be used to deduce mineralogical differences between the concretion and the surrounding rock. The XRD traces show no discernible peaks of calcite and dolomite in the organic core of a concretion, while clear peaks of calcite and dolomite occur in the surrounding calcareous rocks (Supplementary Fig. S3). Only very small calcite and dolomite peaks are seen in the silica concretion. The concentrations of calcite and dolomite in the concretion are therefore apparently smaller than in the surrounding rock. The XRD traces were not calibrated and therefore cannot be used to estimate the absolute quantities of calcite and dolomite in the samples. However, considering the much smaller height of the highest dolomite peak given by the concretion, when compared to the surrounding rocks, the concentration of dolomite in the concretion must be considerably smaller than the concentration in the surrounding rocks. Furthermore, in the case of the surrounding rock the highest dolomite peak is much higher the highest calcite peak, whereas in the concretion these peaks are roughly the same height. These differences indicate that relative to calcite there is a much reduced proportion of dolomite in the concretion compared to the surrounding rock.

This interpretation from the XRD results is consistent with the compositional differences between the concretion and surrounding sedimentary rock as determined by XRF (Supplementary Table S1). The mean molar Ca/Mg ratio is 2.65 in the concretion and 1.22 in the surrounding rock (Supplementary Table S1). The CaO contents of the concretion and surrounding rock are 9.95 wt% and 22.26 wt% respectively, while the MgO contents of the concretion and surrounding rock are 2.70 wt% and 13.09 wt% respectively. These differences can be explained by calcite and dolomite having lower concentrations in the concretion than in the surrounding rock, but the ratio calcite/dolomite being larger in the concretion than in the surrounding rock.

Optical observation of thin-sections revealed that euhedral quartz crystals are distributed in the bitumen cores. However, very fine quartz grains form a dense silica enriched zone (i.e. silica concretion) around the bitumen core (Figs. 1b-3,c-1, 2) and separate it from the surrounding fine-grained calcareous (calcite and dolomite filling) sedimentary rock (Figs. 1b-1, 2).

In the bitumen core, a dense accumulation of microscopic organic spheres (micrometres to decamicrometres in size) is observed (Fig. 1d-1). Spherical green algae ‘*Botryococcus buanui’* have been widely reported from the Green River Formation^41^, and the observed organic spheres probably originated as green algae^42^. Within the same part of the Green River Formation, many fish coprolites containing similar highly concentrations of spherical organic algae have been identified (Figs. 1d-2, 3). This suggests that the origin of the bitumen cores^43–46^ is coprolite containing ‘*Botryococcus buanui’*^47^. The δ^13^C, elemental contents of N, S, and C, and carbon preference index (CPI) of the bitumen cores, silica concretion and surrounding rock were analysed (see “Methods” section). High contents of total organic carbon (ca. 10 wt%) and nitrogen (ca. 0.2 wt%) in the bitumen core have been measured, and the δ^13^C_carb_ and δ^13^C_org_ of the bitumen core, concretion and the surrounding rock have been determined (Supplementary Table S2). Both δ^13^C_carb_ and δ^13^C_org_ are lower in the bitumen cores and silica concretion than in the surrounding rocks. TOC is very high in the bitumen cores, high in the surrounding rocks and low in the concretion. N-paraffin analysis also indicates that bitumen cores have relatively high contents of C, N, and S in the organic fraction, with high maturity shown by the CPI^48^ (Supplementary Table S2). The δ^13^C_carb_ value of the concretion (average 0.87‰) could be explained by the inorganic carbon in the silica concretion being about 9.3% derived from the organic carbon in the core (− 28‰) and about 90.7% derived from sedimentary carbonate^40^ (3.85‰) (Supplementary Table S2).

SXAM mapping has allowed one-dimensional elemental profiles of Si, Ca and other elements to be produced along sections perpendicular to the concretion rim (Figs. 2b-1–c-2, Supplementary Fig. S4). This mapping revealed that Si is concentrated in the concretion while Ca is concentrated in the surrounding rock. XRF analysis also shows contrasting Si contents between the concretion (up to 78 wt%) and the surrounding sedimentary rock (ca. 25 wt%) respectively (Supplementary Table S1).

## Discussion

### Formation process of ellipsoidal silica concretions

Any model for the formation of these concretions must explain several key features: (1) the accumulation of silica around the bitumen cores; (2) the almost uniform concentration of SiO_2_ between the bitumen cores and the rims of the concretions; (3) the steep SiO_2_ concentration gradient across the rims of the concretions, from higher concentrations internally, to much lower concentrations externally; (4) very fine and almost uniform grain size (c. 10 µm) and crystal morphology of silica between the bitumen core and the concretion rims; (5) chemical zoning, with zones parallel to the margins of the concretion; (6) lower concentrations of calcite and dolomite, but higher calcite/dolomite ratios in the concretion than in the surrounding sedimentary rock; (7) carbon isotopic signatures of carbonate samples from between the bitumen core and the rim of the concretion that are intermediate between those of the bitumen and carbonate in the sedimentary rock surrounding the concretion; (8) deviation of sedimentary layers in the surrounding rock around the concretions; and (9) the ellipsoidal shapes of the concretions.

The following process of ellipsoidal silica concretion formation can explain these features. Firstly, many pellets formed from green algal accumulations (presumably ‘Fish coprolite’)^26,46,47^ distributed in the lake sediment (Fig. 3a). After burial in the calcareous lake sediments, which contained alkaline (pH > 9) pore-water, the organic matter in the pellets started to decompose^38,39^. This decomposition produced relatively lower pH pore-water in and around the algal matter (pH < 3–4 in the organic matter; Fig. 3b)^49,50^. The solubility of silica in water increases markedly with increasing pH above c. 9.5. The solubility of amorphous silica is an order of magnitude lower at pH 9.5 than at pH 11 in both fresh water and brine^51^ Acidity diffused out from the bitumen and caused the solubility of silica to decrease, leading to precipitation of silica from the pore water. A precipitation front was formed where silica became over-saturated. Across this front a Si concentration gradient developed, with Si concentration decreasing from the surrounding calcareous sediments towards the surface of the decomposing pellet (which eventually formed the bitumen core), owing to the concentration of organic acids increasing with decreasing distance to the pellet. This concentration gradient caused Si to diffuse towards the decomposing algal pellet, effectively supplying additional Si from the outside (Fig. 3c).

In the proposed model, the precipitated silica fills both pre-existing pores and porosity generated by the acid dissolving the carbonate in the surrounding sediments. Concretion growth by diffusion of acid porewater from the decaying organic core can explain the observations whatever the proportion of primary and secondary porosity are filled by silica.

Silica precipitation in open pore space would have contributed to a decrease in the concentrations of pre-existing dolomite and calcite. However, this dilution process alone cannot explain the apparent changes in calcite and dolomite concentrations, because it does not account for the different calcite/dolomite ratios in the concretion and in the surrounding rock. Possible explanations for these different ratios are: (1) initial dissolution of dolomite and calcite during concretion formation, followed by late diagenetic precipitation of calcite in the small amount of residual pore space within the concretion; (2) formation of the concretion within calcite-rich sediment, followed by dolomitization of the surrounding sediment or sedimentary rock (depending on whether the sediment had lithified) and possibly precipitation of primary dolomite, with only a small amount of dolomite forming in the concretion owing to the low interconnected porosity of the concretion. Dedolomitisation of residual dolomite within the concretion is an unlikely explanation since this process would require elevated Ca/Mg ratios in the concretion compared to the surrounding rock and there is no obvious mechanism by which such ratios could occur.

The XRF analyses of paired samples from the concretion and the immediately adjacent surrounding rock show that the mean Al_2_O_3_ concentration in a concretion on a mass basis is about 28% of that in the surrounding rock, with a range between 24.8 and 30.5% (XRF analyses, Supplementary Table S1). As Al_2_O_3_ is probably present in minerals that are much less soluble in water than carbonate minerals, it can be assumed that this difference in Al_2_O_3_ concentration is due mostly to dilution by precipitated silica. To achieve this dilution would require the initial intergranular porosity to be between 70 and 75%.

If the initial porosity was 75% and it was filled with precipitated silica, the resulting overall silica concentration due to pore-filling silica would be about 70 wt%. However, adding the silica in the original carbonate-dominated sediment, which is ca. 5 wt% of the silica in the concretion, accounting for the assumed 75% initial porosity, gives a total silica concentration similar to the maximum analysed silica concentration in a concretion of 78 wt%.

Primary porosities as high as 75% are feasible only if the concretions formed very close to the sediment–water interface (c.f. compaction data for carbonate sediments in Fabricus^52^; Bjørlykke et al.^53^). On the other hand, release of acid from the organic matter must have dissolved at least some carbonate minerals. The isotopic data are also consistent with some carbon from the organic matter being incorporated into the residual carbonate minerals within the concretion.

The precipitated silica occluded the porosity and reduced the diffusive fluxes of solutes between the surrounding rock matrix and the concretion. Furthermore, carbonate dissolution results in pH increase and a decrease in the degree of oversaturation of silica, with a consequent decrease in the rate of silica precipitation. Carbonate mineral dissolution caused a reduction in the solid Ca and C concentration between the organic core and the concretion rim, as shown in Ca profiles across a concretion and TOC (Figs. 2c-1, 2, Supplementary Table S1). However, porosity generation by carbonate dissolution increases the diffusivity and hence supply of acid and silica, while porosity occlusion by silica precipitation reduced the diffusivity, with the opposite effects on acid and silica supply. Overall, there is a complex non-linear coupling between diffusion of organic acids outwards, porosity generation and consumption of acidity by carbonate dissolution, and diffusion of Si inwards with accompanying silica precipitation. These non-linear processes give rise to the chemical zoning seen in the concretions. Isotopic exchange between the carbon in the organic acids and the carbon in the carbonates can explain the carbon isotopic signatures of the residual carbonate phases inside the concretion.

Initially, silica was probably precipitated as a colloidal or gel aggregate of hydrous sodium silicate, and became crystalline over time^54,55^, resulting in the very fine, almost uniform grain sizes seen across the concretions. Although there is a possibility that the euhedral quartz crystals in the cores of the concretions grew during diagenesis, the timing of crystalline quartz growth is not known (Figs. 1c-1, 2). However, the distribution of major elements observed with SXAM and the zoning observed macroscopically suggest the concretions growing outward due to an H^+^ front (reaction front) diffusing from the decomposing algal pellets^26^. During reaction front development, within the front, concentration and precipitation of SiO_2_ continued until the supply of acidity by diffusion from the algal pellet terminated.

Within such fine layered sediments, the horizontal diffusivity of silica parallel to the layers, would have been higher than the diffusivity of silica perpendicular to the layers. This anisotropy could explain the ellipsoidal shapes of the concretions. Such deviation of sedimentary layers in the sedimentary matrix around the concretions is also strong evidence that the concretions became solid at an early stage of diagenesis before compaction caused by burial was complete.

Throughout concretion formation there would have remained at least a small amount of connected porosity through which organic acids and silica could diffuse. At any time prior to the termination of acid production, the pH of porewater would decrease from the rims of the concretion towards its core. Therefore, silica would be less soluble near the core of the concretion than at the rim of the concretion, producing a silica concentration gradient from the concretion rim towards the core. The result is that transport of silica towards the organic pellet could continue throughout the volume of the concretion. That is, there were elements of both diffusive growth, which determined the overall size and shape of the concretion, and pervasive concretion growth as proposed by Raiswell and Fisher^19^.

Bedded cherts in the Green River Formation were also formed by a similar mechanism involving pH changes in a relatively high pH environment. These pH changes occurred during the decomposition of algal organic micro-spheres^28^ and resulted in a ready supply of silica from the pore water within surrounding calcareous sediments. However, the chert is bedded owing to the algal organic matter being almost homogeneously distributed in the sediment beds, unlike in the concretions, in which higher localized concentrations of algal organic matter occur as fish coprolites. Reflecting its lower average concentration, the organic matter of algal micro-spheres in the bedded cherts was completely decomposed, and almost no residual organic matter remains. In contrast the higher localized organic concentrations in the concretions were not entirely broken down.

After deep burial (ca. 200–600 m) the geothermal temperature increased to between 50 and 100 ℃ and produced the preserved bitumen core^32,56^ which could not migrate readily owing to the dense silica concretion. It is noteworthy that the bitumen cores are well preserved within the silica concretions (Fig. 1b-3, Supplementary Fig. S1) compared to bitumen in the surrounding rocks. Although this is a very distinctive example of silica concretion formation associated with organic matter decomposition, other isolated silica concretions or accumulations observed in other places in calcareous sediments^12,57^ are also possibly formed by the same kind of pH buffering process in very early diagenesis. Published organic geochemical studies carried out on other concretions^58,59^ show that n-alkylated and phytanyl arenes were enhanced in the concretions, relative to the host sediment. These findings also support a very early diagenetic (syngenetic) microbial source for alkylated and phytanyl arenes derived from the microbial ecosystem mediating concretion formation. In contrast, aromatic compounds formed by thermal maturation.

### Growth rate estimation by ‘diffusion–reaction cross plot’

During concretion growth, acidity diffused outwards from the organic core of a concretion and a steep pH gradient formed at the concretion surface (rim) due to a buffering reaction between organic acid and SiO_2_(aq). Corresponding to this pH gradient there is a presently-observed solid phase SiO_2_ concentration gradient with characteristic width ‘L’ (Figs. 2, 3). This width ‘L’ does not reflect weathering after concretion formation, since weathering would also affect the surrounding rock, for example by depleting Ca; such weathering effects are not observed in the surrounding rock. The width ‘L’ reflects the diffusion rate of SiO_2_(aq) and rapid SiO_2_ precipitation due to the pH change at the front in the early diagenesis (Fig. 3c).

**Figure 4:**
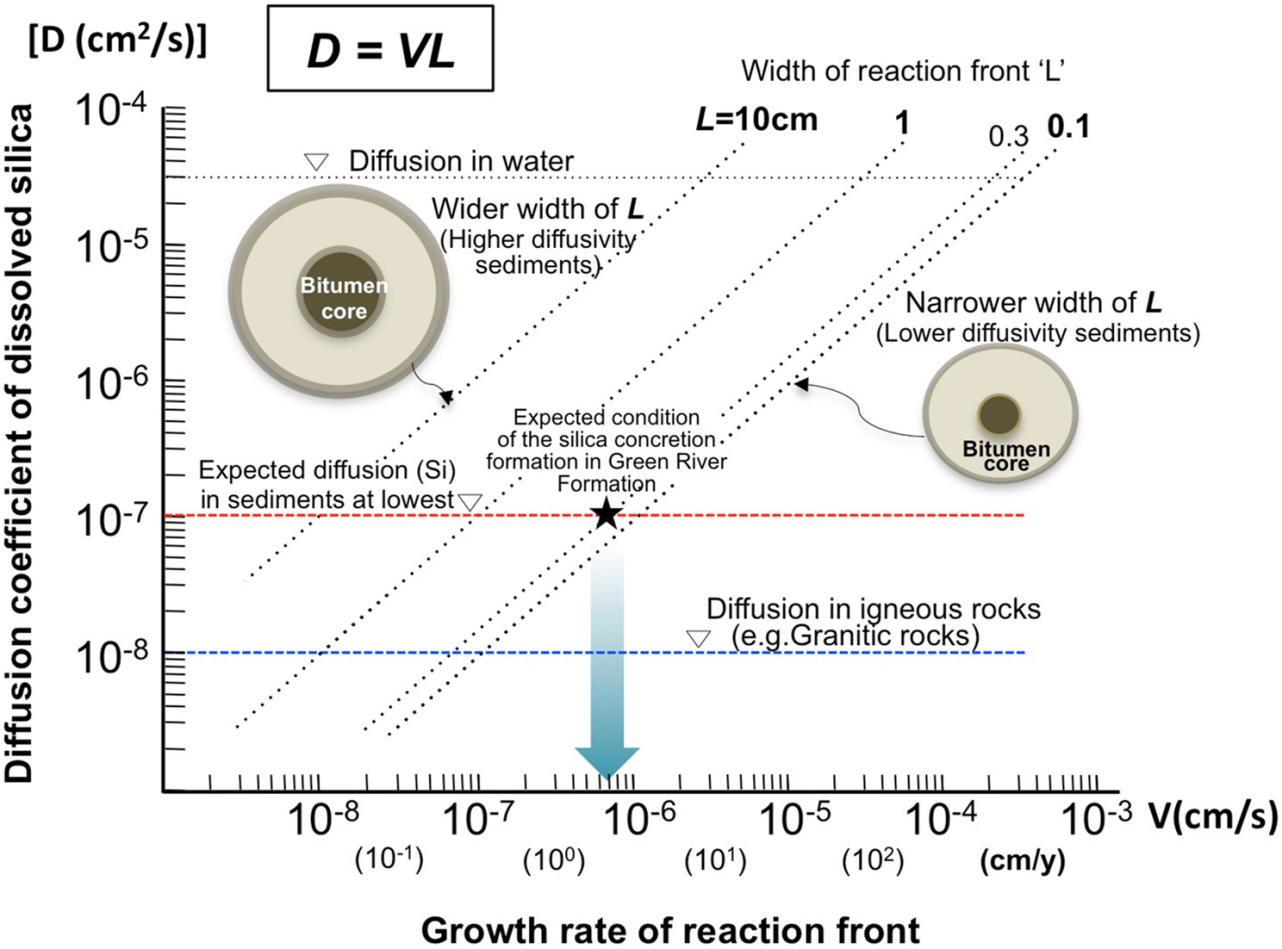
Diffusion–growth rate cross-plot. Relationship between effective diffusion coefficient (D; cm^2^/s) and rate of movement of the reaction front (V; cm/s) defined by dimension analysis. The field over which the silica concretions most likely formed is defined by the width of the reaction front (L = 0.3 cm) and the effective diffusion coefficient of SiO_2_(aq) in similar kinds of marine sediments^60,61^. A very rapid minimum growth rate of the reaction front, in the order of 10^−6^ cm/s, is consistent with the formation of cm sized concretions within 10 years.

The porewater in the surrounding sediment was alkaline, and therefore organic acid from decaying algal matter diffused towards the concretion rim where silica precipitated within the reaction front ‘L’. The width of the reaction front ‘L’, diffusion coefficient of SiO_2_(aq) ‘D’ and concretion growth rate ‘V’ have a relationship: ‘L = D/V’ that can be shown on a simple diagram (Fig. 4)^18,20^.

The acid diffusion timescale required to generate the width L is L/V. At the same time, silica ions diffuse inward, towards the decomposing organic matter from the surrounding sediments outside the concretion. The saturation index of silica in the sediments drastically increases at a front some distance from the organic matter, due to pH buffering shown by the following chemical equations.

1 $${\text{Si}}\left( {{\text{OH}}} \right)_{{3}} {\text{O}}^{ - } + {\text{ H}}^{ + } = {\text{ Si}}\left( {{\text{OH}}} \right)_{{4}} \left( {{\text{aq}}} \right),$$

2 $${\text{Si}}\left( {{\text{OH}}} \right)_{{4}} \left( {{\text{aq}}} \right) \, = {\text{ SiO}}_{{2}} + {\text{ 2H}}_{{2}} {\text{O }}\left( {\text{l}} \right).$$

Between this front and the decomposing organic material the solubility of silica is much reduced compared to greater distances from the organic material than the front. The precipitation threshold is exceeded at the front and Si precipitates there, causing the front to move outwards, away from the core. The time required to exceed the saturation threshold is given by the diffusion timescale of width L, given by L^2^/D. Equating these two timescales leads to:

3 $${\text{L }} = {\text{ D}}/{\text{V}}.$$

The penetration rate (V) at any time would be constant in a fine sediment, since this sediment is homogeneous and there is a negligible temperature gradient across it. Although the diffusion coefficients would be slightly decreased due to the silica precipitation in the sediments, the width ‘L’ should change due to variations in the diffusion coefficient of SiO_2_(aq) in the matrix of sedimentary rocks. This means that a wider L is developed if the matrix has a higher value of D and a narrower L is produced where the matrix has a lower value of D. From the presently observed ‘L’ from the SXAM measurement, we can determine L (= 3 mm). The growth rate of the silica concretion can be used to estimate the value of each ‘L’. A growth timescale τ is given by:

4 $$\tau \, = {\text{ R}}/{\text{V}},$$

where R is the radius of a silica concretion.

From Eqs. (3) and (4), we can constrain the diffusion rate of relevant ions through the calcareous sediments, and the timescale taken for a silica concretion to form, as shown in a ‘Diffusion–reaction rate cross-plot’ (Fig. 4). Published values of effective Si ion diffusion coefficients (in the order of 10^–7^ cm^2^/s) in fine sedimentary rocks with similar characteristics to those studied here^60,61^, can be used to estimate the minimum formation rate of ‘L’. If the value of 3 mm is used, the expected formation time of ‘L’ would be within several years. Therefore silica concretions with sizes of a few cm would have formed within 10 years. Although this diagram can be applied only to etimate the rate of reaction front development, such rapid silica concentration and precipitation is also consistent with estimates based on studies of petrified wood^62^ as well as experimantal work to demonstrate the silicification of trees^63^. The timescales may provide a new perspective for interpreting formation of silica concretions in sedimentary rocks.

## Conclusion

The formation of ellipsoidal silica concretions with bitumen cores, found in the Green River Formation of Utah, can be explained by outwards diffusion of organic acids from coprolite precursors of the bitumen, and inwards diffusion of silica from the surrounding calcareous sediments, accompanied by pH-buffering due to carbonate mineral dissolution. The rims of a concretion represent the limit at which the pH was low enough to cause silica to become sufficiently supersaturated to precipitate. However, the model does not preclude pervasive precipitation of silica throughout the pore space. Since at any time pH would have decreased towards the bitumen core from the concretion rim, the solubility of silica would also have progressively decreased towards the core. This would have caused a diffusive flux of silica inwards, throughout the residual porosity in the concretion, which would never have been completely sealed. Complex non-linear coupling between the transport and mineral precipitation/dissolution reactions produced observed chemical zoning in the concretions. The precipitated silica probably filled both primary pore space and pore space generated by dissolution of pre-existing carbonate minerals.

A ‘Diffusion–reaction rate cross-plot’ shows that the solute transport history during silica concretion growth was of rather short duration and led to the preservation of bitumen in the concretions’ cores. Parametric analysis of transport processes shows that silica concretions are only formed when diffusion occurs in combination with relatively rapid silica precipitation by pH buffering within a time scale of up to a few years for concretions with diameters in the order of cm. The cross-plot also shows the conditions that are most appropriate for the formation of ellipsoidal silica concretions in early diagenesis.

## Methods

First of all, the microscopic occurrences of the silica concretions and bitumen cores were observed in thin-sections by optical microscope and fluorescent microscope. Then, the microscopic textures of the concretions (between the bitumen cores and the surrounding sedimentary rocks) were analysed by SEM with an electron beam of 15 kV/1.75 A (TM-3000, Hitachi Co.). The mineralogy of the concretions and their cores were determined by X-ray diffractometer (XRD; Multiflex, Rigaku Co.) using crushed and powdered samples and Cu-Kα radiation (the Cu being subjected to an electron beam of 20 kV/20 mA).

Concretions were also sliced to produce plane surfaces through their bitumen cores. The spatial distributions of elements across these surfaces were then measured, the measurements spanning both the concretions and surrounding sedimentary matrices. In particular, the 2-D spatial distributions of Si, Ca and other chemical constituents in these planes were carefully analysed semi-quantitatively by X-ray fluorescence analyzer (SXAM). The results were used to determine the widths of the edges (L) of concretions quantitatively. ‘L’ are the widths of the concretions’ thin marginal layers, or ‘reaction front’s’. A value of ‘L’ is the distance along a SiO_2_ concentration profile from the closest point to the core of a concretion where there is a background concentration, to the first point within the concretion where the highest SiO_2_ concentration is attained. The width of a concretion’s margin (L) should reflect the rate of growth and reaction as well as the overall formation time of the concretion in the sediments after burial. To measure L, the SXAM analyses were carried out using an X-ray fluorescence analyzer (XGT-5200V Horiba Japan) at Nagoya University Museum, Aichi, Japan. A high-intensity continuous X-ray beam (Rh anode 50 kV 1 mA), 100 μm in diameter, was focused with a guide tube and irradiated the surface of the sample perpendicularly. The sample was placed on a PC-controllable X–Y stage. X-ray fluorescence from the sample surface was analyzed with the hp-Si detector of an energy-dispersion spectrometer^64^.

XRF analyses were undertaken to measure the contents of Ca and other major elements using a Rigaku ZSX Primus II equipped with a Rh X-ray tube in the Graduate School of Environmental Studies, Nagoya University, Japan. Glass beads were prepared by mixing a portion of each sample, which was ignited at 950 °C to decompose carbonates, with anhydrous lithium tetraborate flux and then fusing. Measurements were calibrated with rock reference samples issued by the Geological Survey of Japan (GSJ: Geochemical Reference Sample Data Base, GSJ: https://gbank.gsj.jp/geostandards/welcome.html). The estimated analytical uncertainties were 1% to 2% for SiO_2_ and CaO and 5% for other major elements^18,20^.

Carbon, nitrogen, and sulfur contents of concretion samples were measured with an elemental analyzer (Elementar Vario EL cube in Nagoya University). Measurements were made on 10 mg powder samples wrapped in tin capsules. We used sulfanilamide (NH_2_C_6_H_4_SO_2_NH_2_) as a standard for the measurement of C, N, and S contents (wt%). Measurement errors are several percent. The data are given in percentage by weight of bulk samples.

The δ^13^C of the concretions were also examined to determine the origin of the carbon in the carbonate. In this study, total carbon (TC), total inorganic carbon (TIC), and total organic carbon (TOC) fractions of concretion samples were analyzed. For TC analysis, about 10 mg of powdered samples were wrapped in tin capsules, and combusted in an elemental analyzer (Themo Fisher Scientific Flash EA1112). The CO_2_ gas evolved in the reaction was then carried into an isotope-ratio mass spectrometer (IR-MS; Thermo Fisher DELTA V Plus) in a He carrier gas and δ^13^C was measured. For TOC analysis, separated sub-samples were leached three times with 2 mol/L HCl following washing by Milli-Q water in an ultrasonic bath at room temperature, and about 10 mg of the residue was wrapped in tin capsules, and measured δ^13^C by the same method as above^18^.

For TIC analysis, about 20 mg of each sample was reacted under vacuum with 5 mL of 100% phosphoric acid under vacuum over night at 80 °C. This process extracted CO_2_ evolved from carbonate in the sample. The CO_2_ extracted was collected and purified cryogenically. Next, the sample-acid mixture was centrifuged and the residue was washed repeatedly with Milli-Q water to remove all phosphoric acid before being lyophilized. About 10 mg of the residue was combusted with CuO in a sealed vacuum quartz tube at 850 °C for 3 h to produce CO_2_. The TOC gas extracted was then cryogenically purified to CO_2_ by removing H_2_O using ethanol slush traps (ca. − 90 °C) and liquid nitrogen traps under a vacuum line. The CO_2_ was measured for δ^13^C by an IRMS with a dual inlet system (Delta-V Advantage; Geological Survey of Japan). All of the δ^13^C data were represented with respect to Vienna Pee Dee Belemnite (VPDB) standardized by NBS-19. The measurement errors were less than ± 0.1‰ for δ^13^C^18,20^.

N-paraffins in each 2 g sample were extracted twice by ultrasonication with 10 mL of dichloromethane. The extract was applied to a silica gel column with activated 0.5 Al_2_O_3_ and 2.0 g silica gel. A fixed amount of mixed standard (C_11_H_23_COOCH_3_, C_16_H_33_COOOCH_3_, and C_28_H_57_COOCH_3_) was added to the n-paraffin fraction eluted by 5 mL hexane. The fraction was analyzed by flame ionization detector- gas chromatograph (FID-GC; Shimadzu GC-2014 in Nagoya University). FID-GC analysis used helium as a career gas and equipped HP-5 column (Agilent Technologies, 30 m × 0.25 mm i.d., 0.25 µm film thickness). The temperature program was from 50 °C for 3 min to 225 °C at 3.5 °C/min., and 325 °C for 10 min at 5 °C/min. The identification of each organic compound was based on relative retention times against dodecyl-, octadecyl- and triacontyl-acetate obtained by FID-GC.

## Supplementary Information

Supplementary Information.
