# Supplementary material for: Syngenetic rapid growth of ellipsoidal silica concretions with bitumen cores
Source: Sci Rep. 2021 Feb 19;11:4230. doi: 10.1038/s41598-021-83651-w (PMC7895967; doi:10.1038/s41598-021-83651-w)

**Syngenetic rapid growth of ellipsoidal silica concretions**

**with bitumen cores**

Hidekazu Yoshida^1*^, Ryusei Kuma^2^, Hitoshi Hasegawa^3^, Nagayoshi Katsuta^4^, Sin-iti Sirono^3^,

Masayo Minami^5^, Shoji Nishimoto^6^, Seiji Kadowaki^1^, Natsuko Takagi^7^ & Richard Metcalfe^8^

1) Nagoya University, University Museum, Chikusa, Nagoya, Japan

*corresponding author) dora@num.nagoya-u.ac.jp

2) Graduate School of Environmental Studies, Nagoya University, Chikusa, Nagoya, Japan

3) Faculty of Science and Technology, Kochi University, Kochi, Japan

4) Faculty of Education, Gifu University, Yanagido, Gifu, Japan

5) Institute of Space-Earth Environmental Research, Nagoya University, Nagoya, Japan

6) Nagoya City Science Museum, Nagoya, Japan

7) Faculty of Science, Nagoya University, Nagoya, Japan

8) Quintessa, Videcom House, Newtown Road, Henley-on-Thames, Oxfordshire, UK

**Supplementary data**

**(Mineralogy and geochemistry of silica concretion)**

Silica concretions with bitumen cores from Lower to Middle Eocene lacustrine calcareous silty to muddy deposits of the Green River Formation, Utah were mineralogically and geochemically analyzed, and the data obtained are given here.

**Occurrence of bitumen core, SEM and XRD analysis of a silica concretion**


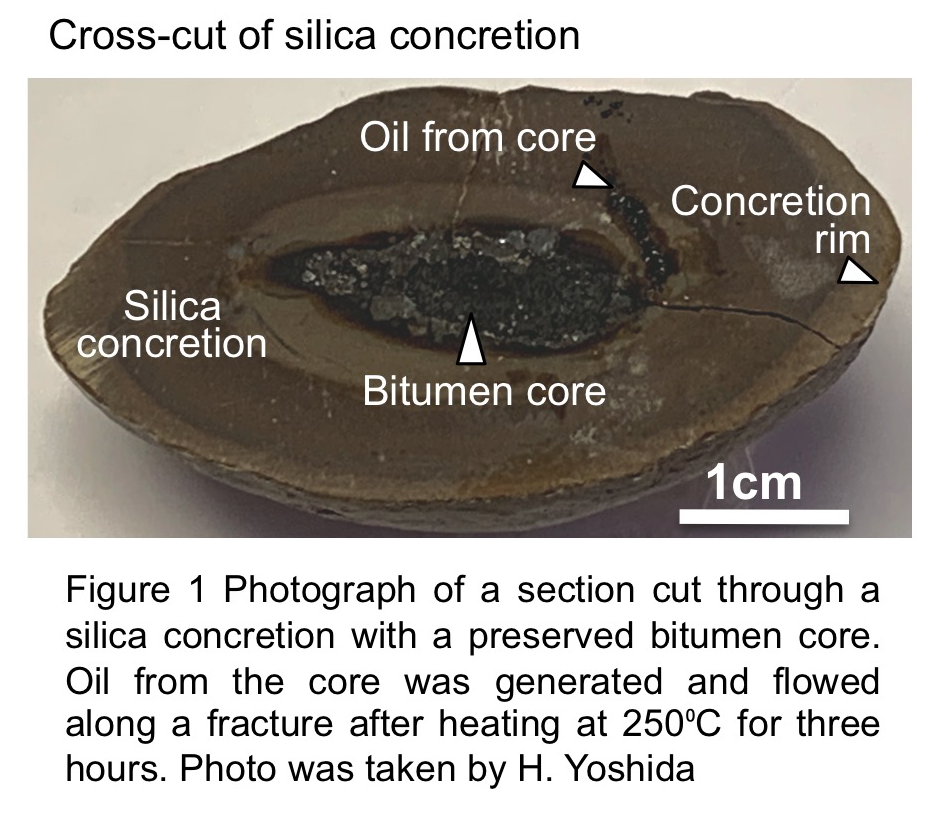
Fig. 1 shows the bitumen core of a concretion (dark brown part) and oil produced by it after heating at 250℃ for three hours. Terms used for different parts of the concretion are also shown in Fig. 1. SEM photomicrographs of sections from a silica concretion and surrounding rock (Figs. 2a ~ d) show very fine dolomite crystals in the surrounding rock and very fined silica crystals in the concretion (all crystals being c. 10 µm across). Quartz crystals in the concretion are equidimensional and show no significant variation in size and shape across the concretion.

Minerals within the bitumen core, concretion and surrounding rock were confirmed with an X-ray diffractometer (XRD; Multiflex, Rigaku Co.) using crushed and powdered samples and Cu-Kα radiation (the Cu being subjected to an electron beam of 40 kV/20 mA). XRD patterns show clear differences between the bitumen core, concretion and surrounding rock (Fig.3). Quartz (Qz) is the dominant mineral in the bitumen core and silica concretion, while the surrounding rock is mainly composed of Dolomite (Do), calcite (Ca) and gypsum (Gy).

**
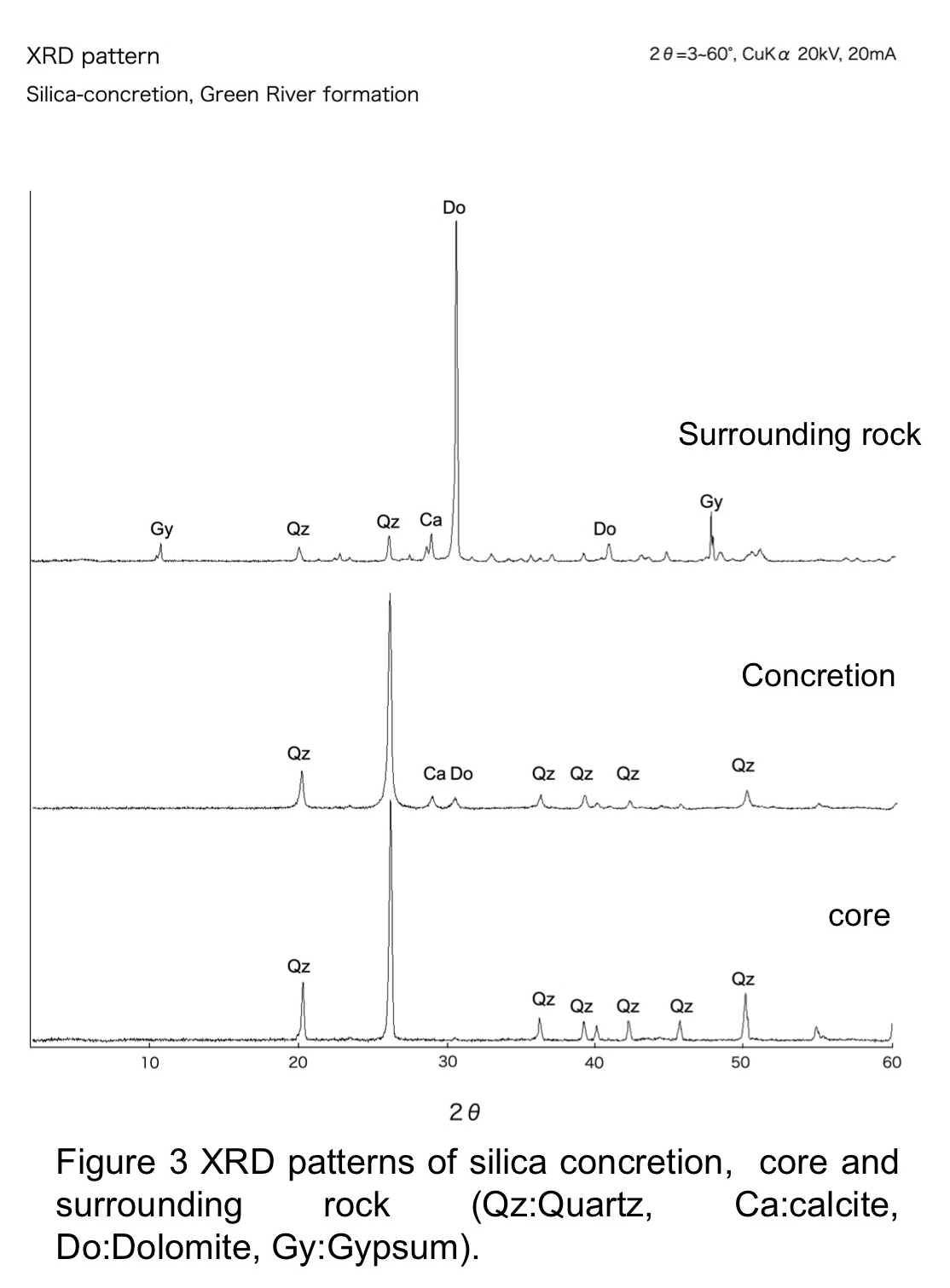

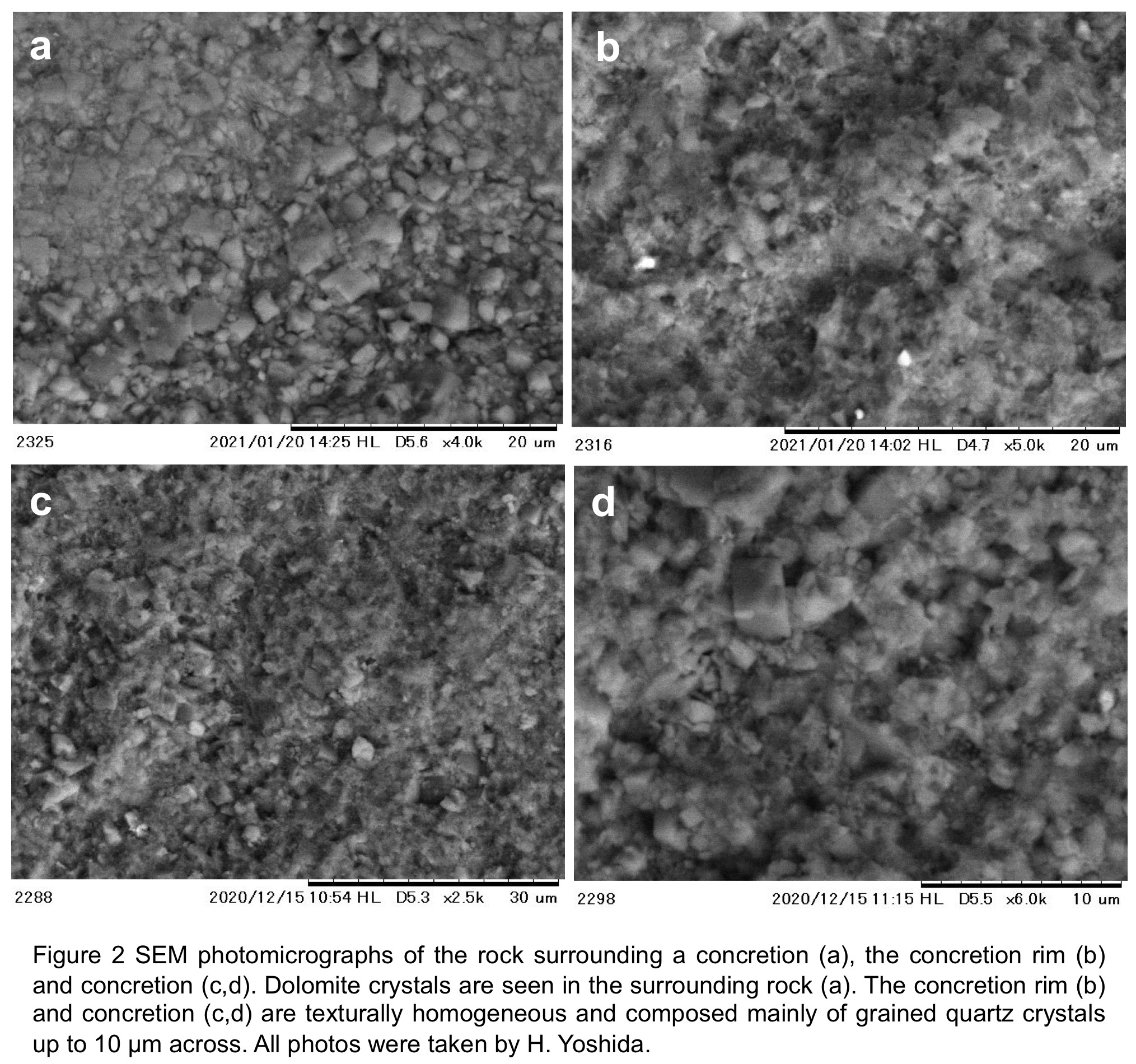
**

**Elemental mapping across silica concretion**

SXAM intensity maps were reduced to one-dimensional element profiles in a direction perpendicular to the concentric pattern identified in a silica concretion (Fig. 4). There is an almost constant Si concentration across the entire width of the concretion, while the Si concentration is markedly lower in the surrounding rock. The Si concentration decreases steeply across the rim of the concretion. Spatial distributions of Fe, K and Sr are similar to the spatial distribution of Ca and possibly reflect temporal variations in lake water chemistry from which the porewater was derived.

**
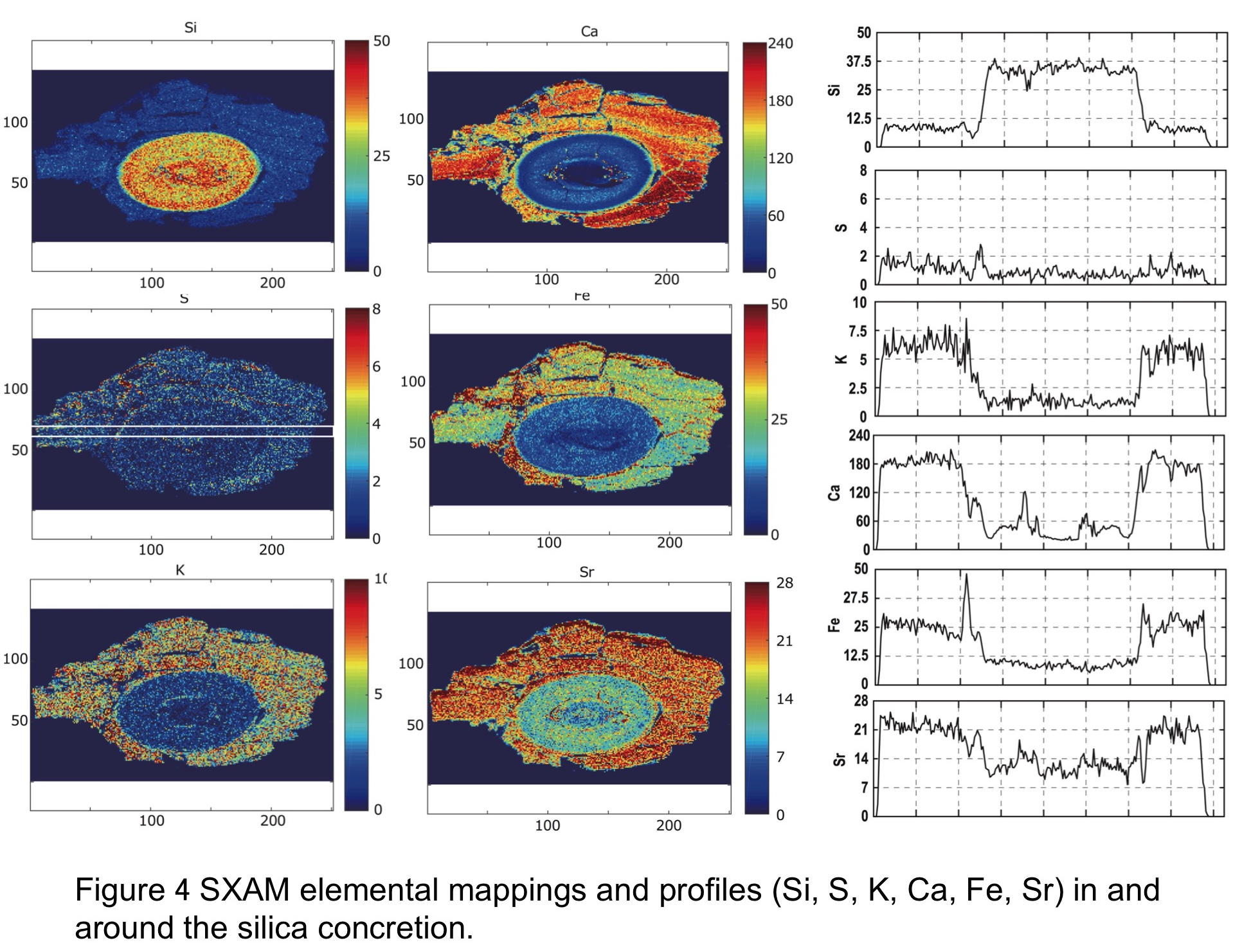
**

**
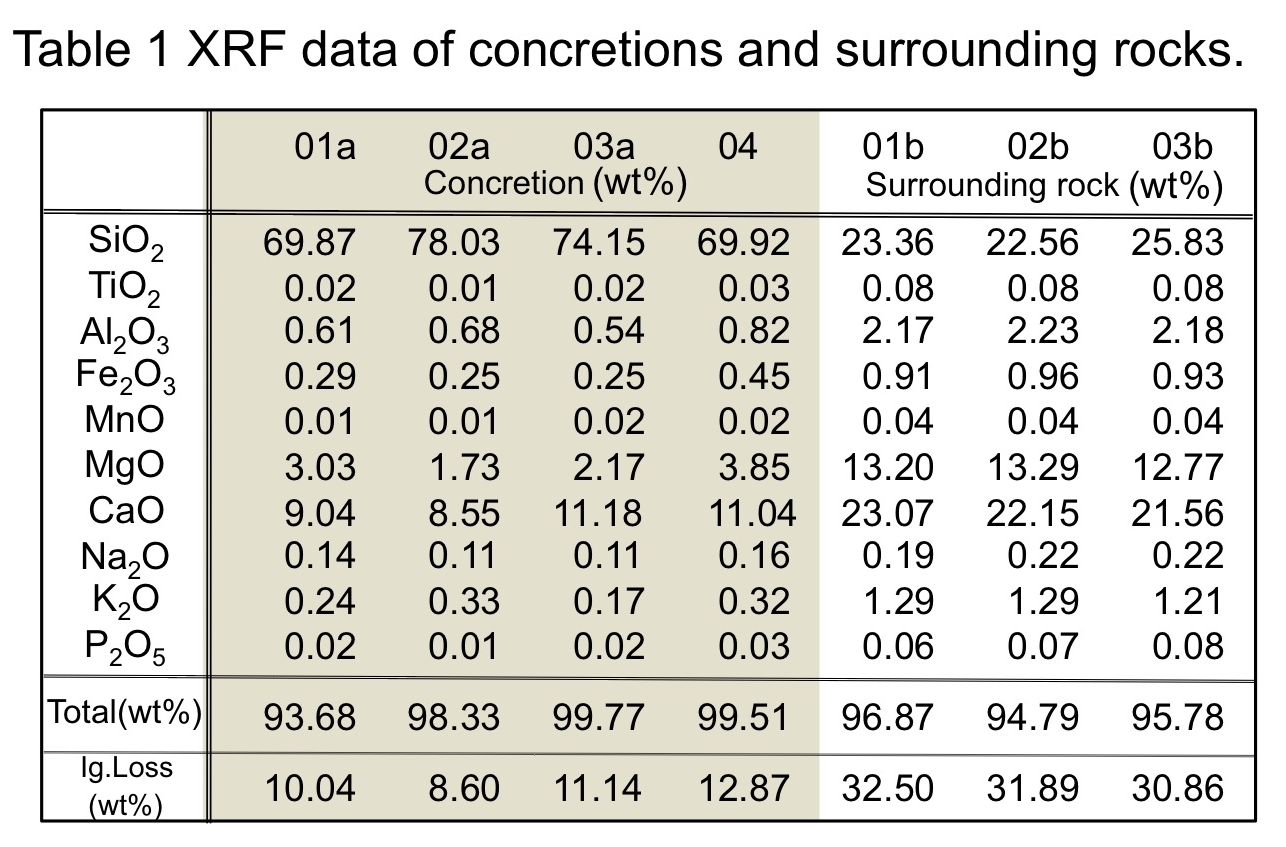
XRF analysis**

Table 1 shows the major element compositions of silica concretions (01a ~ 03a and 04) and associated surrounding rocks (01b ~ 03b). Concretions have relatively high Si contents while Ca concentrations are relatively high in the surrounding rock. The large ignition loss is also consistent with the high content of carbonate in the surrounding rocks.

δ**^13^C value, N, S, and C contents, and Carbon Preference Index (CPI)**

Table 2 shows the measured δ^13^C of TC, TIC and TOC, concentrations of N, S, and C, and CPI of the silica concretions and surrounding rocks. The total organic carbon (TOC) has a markedly negative δ^13^C. The concretion core has relatively high contents of C, N, and S in the organic fraction with a high maturity suggested by the CPI.


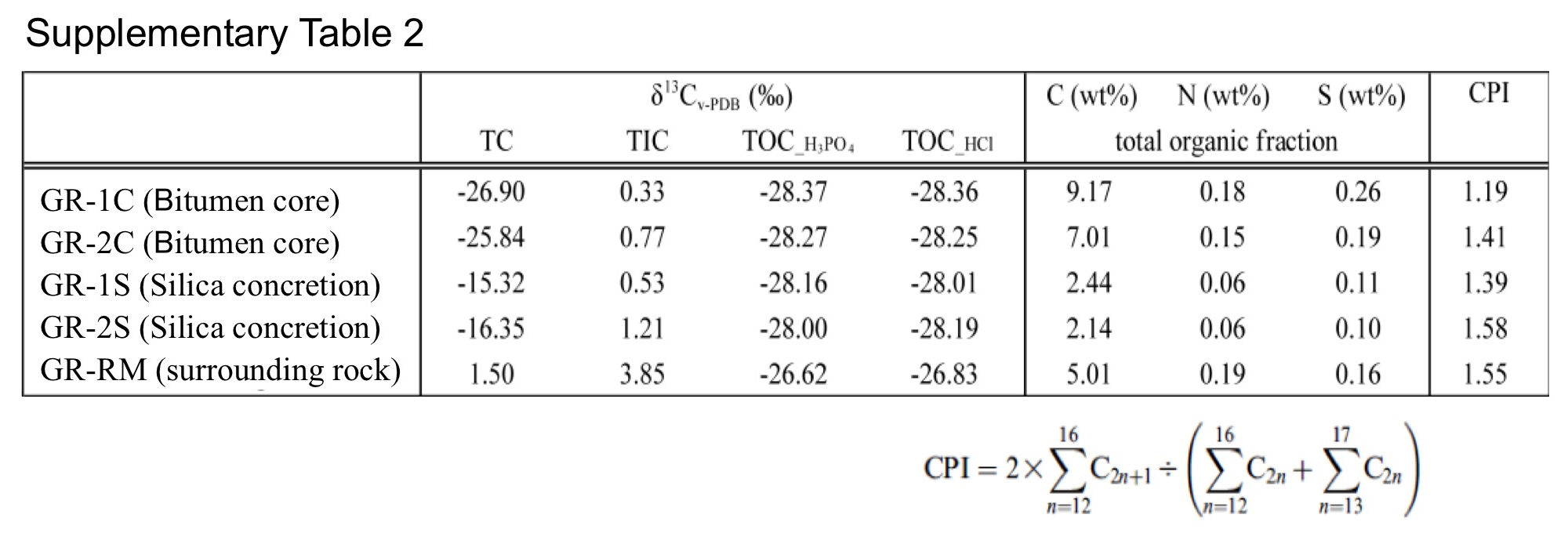

Supplement: Supplementary file 1 — Supplementary Information. [file 41598_2021_83651_MOESM1_ESM.docx]
